# Research Progress on the Regulation Mechanism of Key Signal Pathways Affecting the Prognosis of Glioma

**DOI:** 10.3389/fnmol.2022.910543

**Published:** 2022-07-22

**Authors:** Hao Wu, Min Wei, Yuping Li, Qiang Ma, Hengzhu Zhang

**Affiliations:** ^1^Graduate School of Dalian Medical University, Dalian, China; ^2^Department of Neurosurgery, The Yangzhou School of Clinical Medicine of Dalian Medical University, Dalian, China

**Keywords:** glioma, gene expression, signal pathway, prognosis, neoplastic

## Abstract

As is known to all, glioma, a global difficult problem, has a high malignant degree, high recurrence rate and poor prognosis. We analyzed and summarized signal pathway of the Hippo/YAP, PI3K/AKT/mTOR, miRNA, WNT/β-catenin, Notch, Hedgehog, TGF-β, TCS/mTORC1 signal pathway, JAK/STAT signal pathway, MAPK signaling pathway, the relationship between BBB and signal pathways and the mechanism of key enzymes in glioma. It is concluded that Yap1 inhibitor may become an effective target for the treatment of glioma in the near future through efforts of generation after generation. Inhibiting PI3K/Akt/mTOR, Shh, Wnt/β-Catenin, and HIF-1α can reduce the migration ability and drug resistance of tumor cells to improve the prognosis of glioma. The analysis shows that Notch1 and Sox2 have a positive feedback regulation mechanism, and Notch4 predicts the malignant degree of glioma. In this way, notch cannot only be treated for glioma stem cells in clinic, but also be used as an evaluation index to evaluate the prognosis, and provide an exploratory attempt for the direction of glioma treatment. MiRNA plays an important role in diagnosis, and in the treatment of glioma, VPS25, KCNQ1OT1, KB-1460A1.5, and CKAP4 are promising prognostic indicators and a potential therapeutic targets for glioma, meanwhile, Rheb is also a potent activator of Signaling cross-talk etc. It is believed that these studies will help us to have a deeper understanding of glioma, so that we will find new and better treatment schemes to gradually conquer the problem of glioma.

## Introduction

Glioma is the most common primary malignant tumor. It is characterized by a high recurrence rate. There is a lack of effective treatment strategies, resulting in high mortality and short survival time. Only 5.5% of patients usually survive for 5 years after diagnosis (Ostrom et al., 2020). The median overall survival is approximately 14.5–16.6 months (Wen and Reardon, 2016). According to the classification of the tumors of the central nervous system (by WHO and CBTRUS), brain gliomas are divided into four levels (grade I–IV). Grade IV is characterized by the highest degree of malignancy, the strongest invasiveness, the worst prognosis, and the highest proportion of malignancy. The tumors belonging to this grade are known as glioblastoma or pleomorphic glioblastoma. Glioblastomas, based on gene expression, can be classified into anterior neural type, neural type, classical type, and mesenchymal type (Omuro and DeAngelis, 2013). Although different subtypes have been discovered over the years, there is no precise and effective targeted therapy for glioma that can improve its prognosis (Chen et al., 2017). Moreover, most low-grade gliomas recur, and gene mutations are observed post operation (Fangusaro et al., 2019). The tumor heterogeneity changes, the biological morphology worsens, and abnormalities are observed (Dentro et al., 2021). Inevitably, the generation of pleomorphic glioblastoma linked with high-grade gliomas is observed (Claus et al., 2015).

Despite the tremendous advances in the area of surgery, radiotherapy, and chemotherapy, but the prognosis of glioma still remained considerably unsatisfactory, which could be attributed to its complex and variability of pathogenesis and cellular origin (Xu S. et al., 2020). Therefore, to develop and find efficient treatment methods for glioma, it is significant important to further understand the pathogenesis associated with glioma. Due to the complexity of genetic and environmental initiation events and the lack of clarity of primitive cells or tissues, the initial origin of glioma and the specific correlation and crosstalk of gene mutations and signal pathways in various links (Johnson et al., 2014). Since the beginning of mRNA translation, there have been more complex and variable progress mechanisms (Sellars et al., 2020). Researchers associated with the sphere of basic research and clinical trials have reported that polygenic mutation is the primary mechanism responsible for the occurrence of glioma (Han S. et al., 2020). It is also linked with the processes of development, mutation superposition, and multi-step formation (Zhang et al., 2019). Tumor suppressor genes such as TP53, phosphatase, p16, and phosphatase tensin homolog (PTEN) control the progression of the cell growth cycle, proliferation, and invasion (Suwala et al., 2021). The mutation or deletion of tumor suppressor genes are conducive to regulate the microenvironment of glioma (Calvo et al., 2021). The “6” expression and modification of proteins (phosphorylation, ubiquitination, methylation, acetoxylation, glycosylation, and nitrosylation) can enable them to occur or further deform (Van Meir et al., 2010; Urulangodi and Mohanty, 2020). It has been widely reported that it is the key regulator of numerous GBM cell lines (Ishii et al., 1999). Oncogenes or anticancer genes usually control the malignant behavior of cancer cells by regulating different signal pathways (Liu et al., 2021). This affects the treatment efficiency and prognosis of glioma (Asad et al., 2021). This review describes the key signaling pathways and the associated regulatory mechanisms that affect the prognosis of glioma. The recent findings have been presented herein.

## Hippo/Yes-Associated Protein Signal Pathway

### Origin of Hippo/Yes-Associated Protein Pathway

**FIGURE 1:**
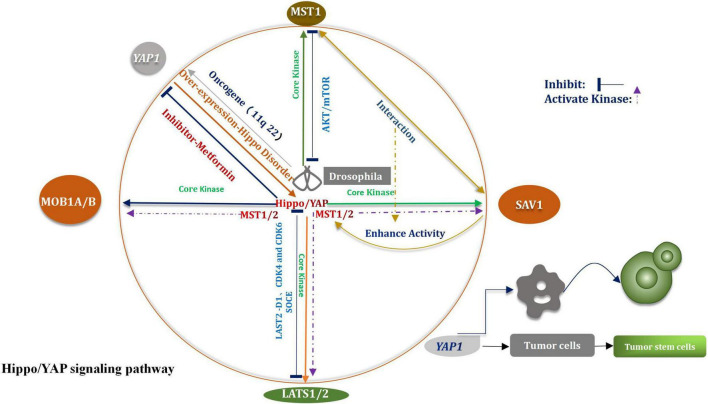
Hippo/YAP signal pathway.

The Hippo signaling pathway was first identified in Drosophila melanogaster (Moloudizargari et al., 2022). This pathway plays a considerable important role in regulating the homeostasis of the tissue environment. The pathway primarily affects the processes of organ development and tumor promotion (Qiao et al., 2018). The key core kinases are Salvador (Sav), Hippo (Hpo), Warts (Wts), and Mob as the tumor suppressors (Mats) (Shimizu et al., 2008). The Hpo–Sav complex can activate the Wts–Mats complex, thereby the downstream effector of the Hippo signaling pathway and the transcription coactivator Yorkie (Yki) can be gradually phosphorylated (Sun X. et al., 2019). Through the study and analysis of evolutionary processes, researchers have found and confirmed that most of the core kinases belong to families of homologous genes, These studies shown that the Hippo signal pathway had not changed in multistage, multi overlap, and multi genes in the evolutionary process, but presents a highly conserved condition (Kim D. H. et al., 2019). According to research findings that the core kinases present in Drosophila melanogaster and mammals are highly homologous (Hong and Guan, 2012). Four types of core kinases, MST1/2, SAV1, LATS1/2, and MOB1A/B, are related with the Hippo signaling pathway in mammals (Juan and Hong, 2016). During the process of regulation, when various upstream signals are received, three types of core kinases (SAV1, LATS1/2, and MOB1) are activated by the activated MST1/2 system, subsequently, they also are phosphorylated (Zhou and Zhao, 2018). The bioactivity of the MST1/2 kinase is promoted under the conditions of the mutual crosstalk between MST1/2 and SAV1 (Bae et al., 2020). Under the above conditions, the expression and function of LATS1/2 are further enhanced. The activated Hippo signaling pathway facilitates the phosphorylation of Yap/TAZ on multiple serine residues, enables it to be degraded independently (Luo et al., 2020), All the above processes are mediated by proteasome. Through the negative feedback, the Hippo signaling pathway regulates the Yes-associated protein (Yap), which is a major of importance transcriptional coactivator (Figure 1; Yan F. et al., 2020). The Hippo signaling pathway is widely correlative a variety of biological processes, such as cell proliferation, apoptosis, tissue repair, and regeneration (Fan et al., 2016). The incidence and progression of varieties of tumors, including glioma, can be potentially traced when the Hippo signaling pathway is abnormally regulated (Johnson and Halder, 2014). Recently, the function of CKAP4 (Cytoskeleton-associated protein 4) on glioma was investigated *in vitro* and in an orthotopic brain tumor model in mice. The results shown that CKAP4 is highly upregulated in glioma and high CKAP4 expressing tumors were associated with poor patient survival. And CKAP4 promotes malignant progression of gliomas *via* inhibiting Hippo signaling (Luo et al., 2021). It has been widely reported that it plays a rather of importance inhibitory role in human cancer and the core key enzymes MST1/2 and LASTS1/2 are involved in the advancement of the Hippo signaling pathway (Xia et al., 2017). It has also been demonstrated that MST1 had a certain impact on glioma, including the process of progression and occurrence of the tumors. Chao et al. (2015) had indicated that the down-regulation of Mst1 can accelerate the proliferation and growth of glioma cells, can also help to inhibit the course of apoptosis. The Akt/mTOR pathway is able to inhibit the proliferation of glioma cells when Mst1 is in over-expression condition (Chao et al., 2015). *In vitro* studies of glioma cells showed that the levels of mRNA and proteins which associated with LATS1decreased significantly (Ji et al., 2012). The migration, proliferation, and aggression of cells were suppressed by the over-expression of LATS1. Liu et al. (2019) indicated that when SOCE was activated, the phosphorylation of LATS1/2 was subsequently triggered, meanwhile, the growth of glioblastoma was also restrained. Other researchers have verified that the proliferation of glioma cells could be potentially stifled by LATS2 (Guo et al., 2019). The path might enable to proceed *via* the reduction in the expression levels of cyclin D1, CDK4, and CDK6. A perspective has also been raised that the way of G1/s transformation is influenced by CDK4, CDK6, and cyclin D1 (Guo et al., 2019).

### Carcinogen Yes-Associated Protein

Yes-associated protein (YAP) plays a key regulatory role in the mediation of numerous malignant tumors (Vargas et al., 2020). It also provided an appropriate and suitable cellular environment for the progression of tumors (Panciera et al., 2020). YAP1 is an oncogene which is over-expressed during the invasion and infiltration of glioblastoma. Guichet et al. (2018) studied 117 glioma samples to analyze the expression of YAP protein in detail. They drawn a conclusion that the expression of YAP was closely relevant to the molecular subtype of glioma and was capable of affecting the prognosis of patients. Some researchers came to conclusion that YAP might be a potentially prognostic indicator of overall survival in patients suffering from gliomas. It is a particular of importance finding for patients suffering from low-grade gliomas. The upregulation of YAP has been widely studied in gliomas (Zhao et al., 2021). Unfortunately, it is not complete clear that whether the specific mechanism is associated with the process. The track of cell transformation can be possibly induced under the conditions of overexpression of YAP (Zhang C. et al., 2021). This way can be beneficial to prevent cell contact inhibition. Attack on host tissues and metastasis may also be fostered under these conditions (Zhao et al., 2007). The role of *YAP1* in the process of proliferation of glioma cells was deeply studied by analyzing shRNA and using over-expression-based methods (Zhang et al., 2016). *YAP1* is a major carcinogen that affects tumorigenesis (Hatterschide et al., 2022). As YAP1 is such an important regulator of tumor progression, its role in glioma expressing IDH1 with an R132H mutation was reported (Patrick et al., 2021). Attenuated nuclear levels of YAP1 in IDH1 mutant glioma tissues and cell lines were concomitant with decreased levels of mitochondrial transcription factor A (TFAM). It is of importance that these findings indicated that bosutinib treatment also aggrandized ROS levels and induced apoptosis in IDH1 wild-type cells when YAP1 was simultaneously exhausted. These findings emphasize the participation of YAP1 in coupling mitochondrial dysfunction with mitochondrial shuttling of TERT to constitute an essential non-canonical function of YAP1 in the regulation of REDOX homeostasis (Patrick et al., 2021). In multiple different types of tumors, including glioma, the amplification of the chromosome zone in the Yap1 gene (11q22) has been discovered (Modena et al., 2006). The over-expression of *YAP1* leads to an imbalance in the Hippo signal transduction pathway (Hayashi et al., 2015). Under the above conditions, it is extremely difficult to control the processes of cell proliferation and tumor growth (Liu and Wang, 2015). An increased extent of nuclear localization and overexpression of *YAP1* has been inspected in numerous cancer tissues (Zhao et al., 2007). Epigenetic changes or mutations result in the inactivation of the Hippo signaling pathway in cancer cells (Cavalli and Heard, 2019; Wu et al., 2019). Under all the conditions, *YAP1* is activated and then is transferred to the nucleus. Cancer cells will be transformed into cancer stem cells when *YAP1* is activated (Schaal et al., 2018). The activation of *YAP1* also promotes occurrence of tumors and aggravates the deterioration and metastasis of tumors (Maehama et al., 2021). In addition, some findings suggested that AMOTL1 (Angiomotin-like 1) may exert a tumor-promoting function in glioma by enhancing the activation of YAP1 signaling. Thus, AMOTL1 may be a potential target for the development of antiglioma therapy (Xu et al., 2021).

To sum up, according to the above results, we speculated that CKAP4 has potential as a promising biomarker and can predict the prognosis of patients with gliomas. And targeting CKAP4 expression may be an effective therapeutic strategy for the treatment of human gliomas. *YAP1*’s up-regulated molecular pathway in glioma is the most feasible molecular target that needs to be broken in the future to improve the treatment processes of glioma. It can also help to promote the development of the area of medical research and provide a point of penetration and breach for the emergence of a new field. The results can potentially help the realm to reach a new peak, meanwhile More clinical patients are benefit from the process. With the further growth in *YAP1* expression, the invasive trait of glioma also raises. The down-regulation of *YAP1* can be potentially helpful to develop new treatment methods for glioma. Furthermore deeper researches, including basal studies and clinical trials, need to be testified if the *YAP1* inhibitor can be a promising therapeutic target. Hence, it is so profound of importance to identify *YAP* inhibitors that to develop new and efficient treatment methods for glioma. Recently, researchers have been focusing on this aspect. It has been recently reported that metformin is a *YAP* inhibitor, and it can be used to treat glioma (Yuan et al., 2018). We believe that efforts are able to accomplish arduous tasks.

## PI3K/Akt/Mammalian Target of Rapamycin Signal Pathway

### Composition and Classification of Phosphatidylinositol-3-Kinase

Serine/threonine and lipid kinases, including phosphomembrane-bound phosphatidylinositol-3 (PIP3) kinases, constitute the phosphatidylinositol-3-kinase (PI3K) family (Stefani et al., 2021; Yoshioka, 2021). These enzymes, along with the downstream Akt and rapamycin targets (mTOR), play considerable important roles in numerous key cellular processes such as growth, differentiation, metabolism, survival, and proliferation and so on (Miricescu et al., 2020; Xu Z. et al., 2020). PI3K enzymes can be classified into class I, class II, and class III enzymes (Yoshioka, 2021). Class I PI3K is one of the most widest considered as aberrant class of enzyme which is associated with cancer (Fruman et al., 2017; Koundouros et al., 2020). It consists of heterodimers of a regulatory subunit and a catalytic subunit (Canaud et al., 2021). Based on the activation mode, it is divided into the 1A and 1B categories (Sun and Meng, 2020; Yue et al., 2021). Class 1A PI3K is activated by many cell-surface tyrosine kinases and consists of catalytic p110 and regulated p85 subunits (Clayton et al., 2022). Class II and III PI3Ks are necessary to maintain normal cell functions, up to now, Both are not yet shown to have carcinogenic effects in human (Bilanges et al., 2019). Acetylgalactosaminyltransferase 2 (GALNT2), the enzyme that regulates the initial step of mucin O-glycosylation, has been reported to play a role in influencing the malignancy of various cancers. However, the mechanism through which it influences gliomas is still unknown. Overall, GALNT2 facilitates the malignant characteristics of glioma by influencing the O-glycosylation and phosphorylation of EGFR and the subsequent downstream PI3K/Akt/mTOR axis. Therefore, GALNT2 may serve as a novel biomarker and a potential target for future therapy of glioma (Sun Z. et al., 2019).

### Downstream Target Akt of PI3K

A dominant product of PI3K is PIP3, which is the phosphorylation form of membrane-bound phosphatidylinositol (Lien et al., 2017). It can initiate a wide range of active signal cascades. Serine/threonine kinase Akt is the major downstream target of PI3K (Liu R. et al., 2020). This is also the primary carcinogenic effector of the PI3K/Akt pathway. The phosphatase and the tensin homolog (*PTEN*) are mainly associated with the dephosphorylation of PIP3 (Lee and Pandolfi, 2020; Chauhan et al., 2021). All mentioned above are Akt’s the main negative regulators. The tumor suppressor gene *PTEN* is lost *via* the processes of somatic mutation or epigenetic silencing, and this process is widely observed in various types of cancers (Álvarez-Garcia et al., 2019). Membrane-bound PIP3 can promote Akt to dock with various kinase targets (Lien et al., 2017). Akt is involved in the indirect activation of mTOR, which is a complex cell growth checkpoint and is affected by growth factor signal transduction and the adenosine monophosphate levels (Kim S. W. et al., 2019). When TSC2 is phosphorylated by activated Akt at ser939 (Moore et al., 2014; Miyazaki and Takemasa, 2017). It dissociates from TSC1, resulting in the activation of mTORC1 (Carroll et al., 2016). A heterodimeric protein complex TSC1/TSC2 was considered as a key hub of signal transduction through which various environmental cues were merged to determine the functional activity of mTORC1 signaling. Growth factor-induced activation of the PI3K/Akt pathway led to the TSC2 phosphorylation at numerous residues (two key points, Ser939 and Thr1462), which are considered as be targeted by Akt. About the mechanism of interaction between TSC1, TSC2, and mTORC1. Some researchers have found that VEPH1 is frequently decreased in cancer, an event associated with poor prognosis of cancer patients. VEPH1 forms a stable trimeric complex with TSC1/TSC2. Loss of VEPH1 decreases TSC2 GAP activity, and relieves Rheb to activate mTORC1, which promotes cancer cell proliferation, invasion, and EMT process. Rapamycin can suppress VEPH1 deficiency induced mTORC1 activation and hepatocellular carcinogenesis (Inoki et al., 2002; Manning et al., 2002; Huang and Manning, 2008). MTORC1 activates translation inhibitors-eukaryotic translation initiation factors 4EBP1 and S6K1 and is sensitive to rapamycin such as mTOR inhibitors (Hua et al., 2019). These are also sensitive to rapamycin-like mTOR inhibitors. With increase the activity of MTORC1, an increase in the extent of mRNA translation, protein synthesis, and cell proliferation was observed (Cho et al., 2021). MTORC2 is the “upstream” of Akt, which directly phosphorylates Akt and participates in the regulation of the cytoskeleton (Cirone, 2021). Inhibition of mTORC1 can result in the activation of the PI3K pathway (Cai et al., 2021). This can be attributed to the negative feedback from mTORC2 that results in the phosphorylation of Akt (Amin et al., 2021). The researchers found that Akt/FoxM1 (Forkhead box M1) signaling pathway-mediated up-regulation of MYBL2 (MYB-related protein B2) fosters progression of human glioma. Down regulation of FoxM1 and MYBL2 by siRNAs induced the cell cycle blocking, apoptosis and EMT (Epithelial-mesenchymal transition) of glioma cells. Furthermore, inactivations of Akt/FoxM1 signaling by Akt inhibitor and siRNA-FoxM1 diminish the expression of MYBL2 in glioma cells. So we concluded that MYBL2 is of importance downstream factor of Akt/FoxM1 signaling to stimulate advancement of glioma, and could be considered as a promising gene for molecular targeting therapy and biomarker for radiotherapy of glioma (Zhang et al., 2017).

### Phosphatase Tensin Homolog Gene: Mutation

**FIGURE 2:**
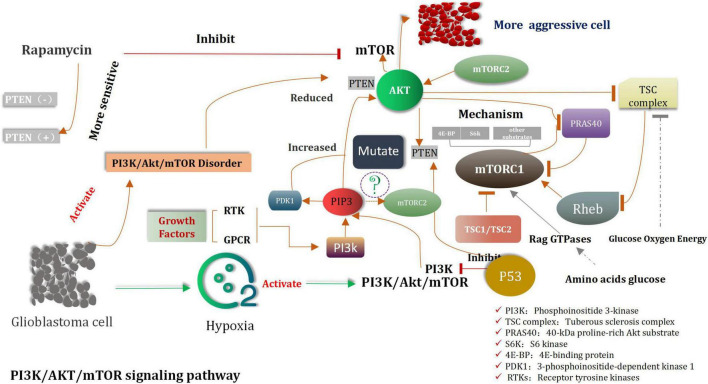
The mechanism of PI3K/AKT/mTOR signaling pathway in glioma.

In gliomas, the PI3K/Akt/mTOR signaling pathway is Anomalously regulated (Liu Z. et al., 2017; Fan et al., 2018; Xu F. et al., 2020; Figure 2). Shortly survival time of patients and strongly invasive features of tumor were associated with decreased *PTEN* levels and increased Akt activity (Zhu and Wei, 2020). In cases of extremely aggressive high-grade gliomas, mutations and the loss of functions of *PTEN* are observed (Mattoo et al., 2019). Cell growth and the expression of PI3K are inhibited by *PTEN*, which is the prime tumor suppressor gene. *PTEN* depletes the PIP3 level, and its loss is directly related with invasive phenotype (Huang W. et al., 2018). The origin of approximately 20–40% of malignant gliomas can be attributed to *PTEN* mutations (Mattoo et al., 2019). *PTEN* mutations are also observed in approximately 65% of high-grade gliomas and 15–40% of primary glioblastomas (Xia and Xu, 2015). It has been previously shown that malignant glioma cell lines containing wild-type PTEN gene can significantly reduce the invasion characteristics cell and migration ability of transfected cells. Rapamycin is a classic mTOR inhibitor (Huang et al., 2019). When these alternative drugs bind to mTOR, they can inhibit kinase activity, and prevent the G1 period of cell cycle to further progress the next step. Preclinical trials were conducted to study and confirm the cell inhibiting effects of rapamycin on glioblastoma and medulloblastoma xenografts (Kuger et al., 2013). However, compared to *PTEN*-positive tumors, the *PTEN* negative tumors are more sensitive to the inhibiting effects of rapamycin (Zeng et al., 2020). Clinical study results revealed that the mTOR and PI3K inhibitors were the candidate drugs which could be used to treat solid tumors in mice (LoRusso, 2016). Highly activated PI3K/Akt/mTOR and the occurrence of the Sonic Hedgehog (SHH) signaling pathways were detected in original cells of glioblastoma. The recent research shown that the combined with inhibitors of PI3K/Akt/mTOR and SHH is obviously better therapeutic effect than the single inhibitor for glioma (Nanta et al., 2019; Zhang et al., 2020).

### Tuberous Sclerosis Complex-mTORC1 Pathway

**FIGURE 3:**
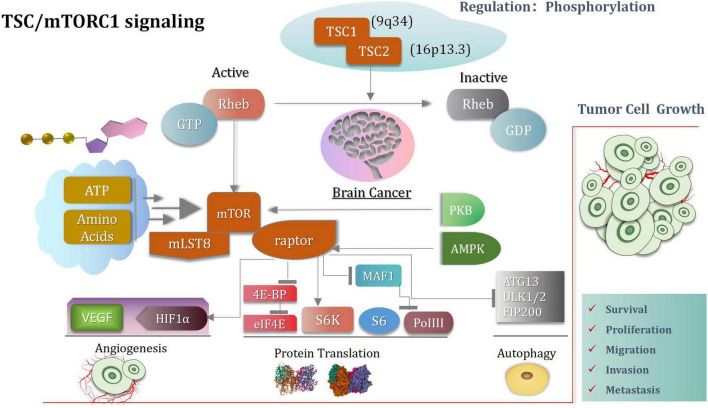
TSC-mTORC1 signaling pathway.

The tuberous sclerosis complex (TSC) complex suppresses mechanistic target of rapamycin complex 1 (mTORC1) (Tee, 2018; Kim and Guan, 2019; Liu and Sabatini, 2020; Prentzell et al., 2021), a central driver of anabolism (Mossmann et al., 2018; Hoxhaj and Manning, 2020). mTORC1 hyperactivity causes diseases related to cellular overgrowth, migration, and neuronal excitability and (Condon and Sabatini, 2019) often arises from disturbances of the TSC complex, consisting of TSC complex subunit 1 (TSC1, 9q34), TSC2 (16p13.3) (van Veelen et al., 2011), and TBC1 domain family member 7 (TBC1D7) (Dibble et al., 2012). mTORC1, consisting of mTOR, raptor and mLST8, controls cell growth mainly through the regulation of protein translation (van Veelen et al., 2011). Activated TSC1:TSC2 complex expresses GTPase activating protein activity toward Rheb, thereby inducing conversion of active GTP-bound Rheb to inactive GDP-bound Rheb. Active Rheb promotes mTORC1 activation, controlling protein translation by activating the ribosomal protein S6 kinase (S6K), inhibiting inhibition eukaryotic initiation factor 4E binding proteins (4E-BP) (Hara et al., 2002; Kim et al., 2002) and inhibiting the RNA polymerase III (PolIII) repressor MAF1 (Kantidakis et al., 2010; Michels et al., 2010). In addition, mTORC1 induces angiogenesis through induction of hypoxia-inducible factor 1a (HIF1a)-dependent expression of vascular endothelial growth factor (VEGF), and inhibits autophagy by phosphorylating ATG13 and ULK1/2 (Hosokawa et al., 2009; Jung et al., 2009). In addition to TSC-dependent regulation of mTORC1 activity, the complex is directly activated by sufficient levels of energy (ATP) and nutrients (amino acids), as well as through phosphorylation of mTOR by PKB and of raptor by AMPK (Vander Haar et al., 2007; Gwinn et al., 2008; van Veelen et al., 2011; Figure 3). Another some researches that Plk1-mediated phosphorylation of TSC1 enhances the efficacy of rapamycin (Li et al., 2018), the results indicates Plk1 activates mTORC1. The researches elucidated that mTOR activity is controlled by two different pathways during cell cycle. In interphase, the PI3K/AKT pathway plays a key role to activate the mTOR pathway by AKT-mediated phosphorylation of TSC2 in response to intracellular signaling. However, in mitosis, Plk1 is the major kinase to activate the mTOR pathway by targeting TSC1. Instead of activation of the mTOR pathway, Plk1 phosphorylation of TSC1 also leads to mitotic defects in an mTOR-independent manner. A novel signaling pathway where Plk1 regulates mTOR independently of AKT in mitosis. The latest researcher indicated that in the context of tumors, low G3BP1 levels enhance mTORC1-driven cancer cell motility and correlate with adverse outcomes in patients. G3BPs are not only core components of SGs (stress granules) but also a key element of lysosomal TSC-mTORC1 signaling (Prentzell et al., 2021).

### The Role of Rheb on mTORC1 Activation

Rheb (Ras homolog enriched in brain) is a member of the Ras superfamily that appears to be conserved in all eukaryotes and, despite the term “brain” in its name, is in fact ubiquitously expressed in mammals (Huang and Manning, 2008). A Rheb homologue exists, named Rhebl1 (Rheb like-1) or Rheb2, that appears to have overlapping functions with Rheb in controlling mTORC1 signaling downstream of TSC1 and TSC2 (Tabancay et al., 2003; Tee et al., 2005). Rheb is a potent activator of mTORC1 (Gromov et al., 1995; Inoki et al., 2003; Yuan et al., 2005). It can stimulate mTORC1, but some researchers suggest that Rheb does not stimulate the activity of mTORC2 kinase (Yang et al., 2006; Huang et al., 2008; Sato et al., 2009). However, active Rheb can indirectly suppress PI3K and mTORC2 signaling by eliciting stimulus varieties of mTORC1-dependent negative feedback circuit systems. Genetic and biochemical studies unified some pathways through identification of two missing links between Akt and mTORC1: the small GTPase Rheb and its negative regulator, the TSC complex, and Rheb is the main mechanism through which PI3K signaling activatesmTORC1 (Goncharova et al., 2002; Castro et al., 2003; Garami et al., 2003; Zhang et al., 2003). Akt inhibits the tuberous sclerosis complex (TSC), the specific GTPase activating protein (GAP) for the small GTPase Once mTORC1 is activated by Rheb, the coinstantaneous phosphorylation of its inhibitory subunit 40-kDa proline-rich Akt substrate (PRAS40) by Akt and mTORC1 itself causes PRAS40 to dissociate from mTORC1. This is thought to increase substrate access to the complex. Glucose, oxygen, and energy levels are also sensed upstream of the TSC complex. Amino acids (and glucose) are sensed upstream of mTORC1 *via* pathways that regulate the Rag GTPases, which do not directly activate mTORC1 but serve to bring it in proximity to Rheb in cells. mTORC1 directly phosphorylates many substrates including ribosomal protein S6 kinase (S6K) and eukaryotic translation initiation factor 4E-binding protein (4E-BP), which mediate its control of anabolic metabolism, cellular growth, and proliferation (Dibble and Cantley, 2015).

### Hypoxia and HIF-1α Promote Cell Proliferation

The induction of hypoxia can activate the PI3K/Akt/mTOR pathway and impulse the migration, expansion, and invasion of the U87 cells (Huang W. et al., 2018). When the inhibitor acts on the PI3K/Akt/mTOR pathway and hypoxia-inducible factor-1α, the migration, expansion, and invasion of the U87 cells were significantly inhibited. Under the same conditions, the expression of HIF-1α can be inhibited by siRNA or the PI3K/Akt/mTOR pathway inhibitors (Xia and Xu, 2015). It can be inferred that tumors exposed to hypoxic environments are often more invasive than those not exposed to such environments. These may evolve into highly malignant tumors (Kim and Lee, 2017). Therefore, these tumors are potentially more resistant to clinical postoperative chemotherapy and radiotherapy. They also exhibit the potential of self-renewal, proliferation, and multidirectional differentiation. It can be further inferred that hypoxia may be related to HIF-1α (Domènech et al., 2021). The combined inhibition of PI3K/Akt/mTOR and the Shh dual pathways and knockout or inhibition of HIF-1α can help reduce the migration ability of tumor cells and prolong the postoperative survival of patients.

In conclusion, the development of drug-resistant tumors presents a significant challenge to the success of conventional treatment approaches. Activation of the PTEN/PI3K/Akt/mTOR pathway is implicated both in the pathogenesis of malignancies and development of resistance to anticancer therapies. Therefore, PI3K/Akt/mTOR inhibitors appear as a promising therapeutic option in association with cytotoxic and other targeted therapies to circumvent mechanisms of resistance. Novel therapeutic strategies could be tailored according to appropriate biomarkers and patient-specific mutation profiles to maximize clinical efficacy and benefit of combination therapies. This will enable administration of selective therapies based on the expression of molecular targets, more appropriately individualizing cancer treatment for patients.

## Long Non-Coding RNAs and miRNA

### Long Non-coding RNA Is a Promising Biomarker

Glioma is characterized by a variety of regulation modes at the RNA level (Lu et al., 2020). Such as miRNA, lncRNAs, and snoRNA, etc. LncRNA is a molecule that also affects the survival ability and various functions of tumor cells (Wang et al., 2020). It is composed of a non-protein-coding unit, the length of which ranges from approximately 200 nucleotides (NT) to > 100 kb (KB) (Hsiao et al., 2016). Thus, LncRNA is a short non-coding RNA. Although IncRNA is emerging in glioma genetics research, it is also a hotspot of current research such as lncRNAOR, lncRNHoxA11-AS and lncRNTUG1. Based on the different genomic positions and backgrounds, lncRNAs is classify as follows: (Jarroux et al., 2017; Lin et al., 2020): sense, antisense, intron, intergenic, and bidirectional (transcribed near the transcription starting point toward the sense and antisense direction) (Ma et al., 2013). As transcriptional regulators, lncRNAs can alter gene transcription through transcriptional interference and chromatin remodeling (that follows the processes of chromatin remodeling and transcriptional interference) (Sarkar et al., 2015). In addition, lncRNA can also regulate and guide translation through the processes of base pairing. It can also regulate the process of splicing by getting combined with splicing factors (Cech and Steitz, 2014). This way can help to change the levels of gene expression after transcription. Results from research conducted in the last 5 years revealed that LncRNAs play an important role in tissue homeostasis and biological processes, including the process of cancer progression (Tan et al., 2021). Recent studies have shown that the epigenetic inheritance, regulation of cell differentiation and cycle of lncRNAs presented abnormal biological behavior, which may affect the occurrence and development of glioma (Plasek and Valadkhan, 2021). Therefore, lncRNAs can be a promising biomarker for the diagnosis, prognosis assessment and targeted therapy of glioma (Bian et al., 2015).

### Long Non-coding RNA

Long non-coding RNAs (lncRNAs) have been shown to be closely related to cancer progression and therapy. Recent demonstrated that lncRNA KB-1460A1.5 inhibits glioma tumorigenesis *via* miR-130a-3p/TSC1/mTOR/YY1 feedback loop. The researchers shown that the lncRNA KB-1460A1.5 is downregulated and positively correlated with prognosis in glioma, and further manifested that over-expression of KB-1460A1.5 inhibits glioma cell proliferation, migration and invasion *in vitro and in vivo*, while down-regulation of KB-1460A1.5 has the opposite effects (Xu et al., 2022). Another some studies have shown that the expressions of HOTAIR1 and CRNDE are positively correlated with the degree of malignancy of tumors, and CRNDE is the most up-regulated lncRNA in gliomas (Seo et al., 2021; Zhang F. et al., 2021). (CRNDE is a long non-coding RNA with alternative splicing and is implicated in the pathogenesis of several cancers) (Jing et al., 2016; Ma et al., 2019). Kaplan-meier analysis revealed that the up-down regulation of BC002811 XLOC-010967 and NR-002809 was significantly correlated with the prognostic survival cycle (Zhi et al., 2015). Another study confirmed that the expression of CRNDE promotes or inhibits the proliferation and migration of cell by phosphorylation of p70S6K (Lu et al., 2018). This suggests that CRNDE can affect the process of mTOR signal transduction (Wang et al., 2015). It has been reported that the expression of MALAT1 in glioma tissue is lesser than normal brain tissue. In the human glioma cell line and glioma xenotransplantation model, the knockout of the MALAT1 gene promotes cell proliferation and invasion (Su et al., 2021). On the contrary, the over-expression of MALAT1 plays a dominant role in the processes of tumor cell proliferation and invasion (Xiao et al., 2020). It can neutralize the significant inhibition of cell proliferation and invasion *in vitro and in vivo*. These results indicated that MALAT1 exhibits tumor-inhibiting properties (Han et al., 2016). In addition, inhibition of the ERK signal (U0126) can simulate the levels of phosphorylated ERK1/2 and MMP2 (induced by MALAT1 over-expression). The results indicate that the tumor inhibitory property of MALAT1 can be attributed to the inhibition of the ERK/MAPK-mediated cell growth and MMP2/9-mediated invading processes (Han et al., 2016).

### MiRNA-Long Non-coding RNA Interaction in Glioma: Research Outcomes

MicroRNA (miR)-mediated mRNA and multiple signaling pathway dysregulations have been extensively implicated in several cancer types, including gliomas. Long non-coding RNA FAM66C regulates glioma growth *via* the miRNA/LATS1 signaling pathway. The luciferase activity of FAM66C was block by miR15a/miR15b, and the promotion of cell growth effects caused by FAM66C deficiency was attenuated by miR15a/miR15b mimics, further proved that FAM66C functioned as a competing endogenous RNA to regulate glioma growth *via* the miRNA/LATS1 signaling pathway (Xiao and Peng, 2021). Based on The Cancer Genome Atlas and Chinese Glioma Genome Atlas databases. The study identified that miR-301a was significantly upregulated in gliomas and was associated with a poor prognosis. Moreover, zinc and ring finger 3 (ZNRF3) exerted a critical role in the miR-301a-mediated effects on the malignant phenotype, such as by affecting proliferation and apoptosis. Mechanistically, ZNRF3 was a direct functional target of miR-301a (Sun et al., 2021). Another study shown that lncRNA KCNQ1OT1 promotes proliferation and invasion of glioma cells by targeting the miR-375/YAP pathway. The results indicated that KCNQ1OT1 was upregulated in glioma tissues compared with adjacent tissues, which was associated with poor prognosis (Ding et al., 2020).

**FIGURE 4:**
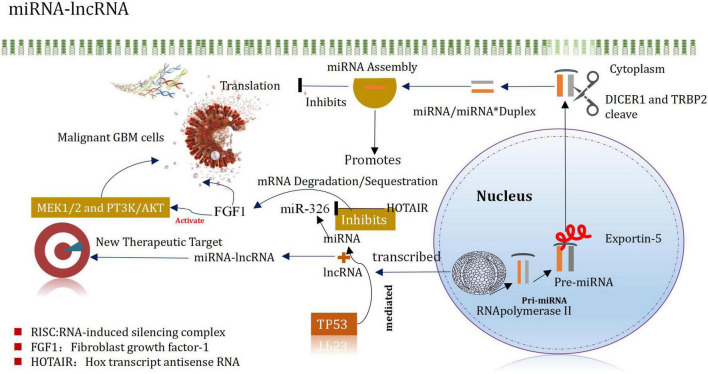
Interaction of miRNA and lncRNA in glioma.

MiRNAs are small non-coding RNA molecules that silence target mRNA through post-transcriptional mechanisms and can leapfrog regulate multiple mRNAs (Mishra et al., 2016). For example, miRNA-21 is highly expressed in GBM, after being inhibited, it can promote the apoptosis of tumor cells (Labib et al., 2020). It plays an important regulatory role in normal cells and tumor cells. Following the processes of transcription, shearing, assembly, silencing, and recombination, they finally recognize the target mRNAs by base complementation. These can degrade the silencing complex or block the further translation of the target mRNA (Kim H. et al., 2017). These can also regulate the expression levels. The miRNAs helps to promote the incidence and development of glioma (Zhang et al., 2017). They also act as a cancer-promoting factor/tumor suppressor factor. It has been reported that miRNAs are characterized by different expression patterns and functional significances in different types of cancers (including glioma) (Godlewski et al., 2015). MiRNAs regulate GSCs, invasive properties, pathogenesis, epigenetic, and signaling pathways (Vinchure et al., 2019; Jiang et al., 2020). MiRNAs have different expression patterns and functional implications in many cancers, including gliomas. MiRNAs are important regulators of GSCs in maintaining aggressive pathogenesis and epigenetic and signaling pathways. Evidences from the new study also indicates that miRNA and lncRNA are associated in controlling the occurrence and development of glioma, and proposes that the mirNA-lncrNA interaction may provide new insights into the therapeutic targets of glioma (Mu et al., 2020; Figure 4). The expression of Mir-148b-3p, a member of the mir148/152 family, is poor in several tumor cell lines. In addition, the research shown that Mir-148B-3p binds to HOTAIR in a sequence-specific manner (Wang et al., 2016a). The down-regulated expression of Mir-326 in glioma specimens and glioma cell lines (U87 and U251) was negatively correlated with the pathological grade of glioma (Ke et al., 2015). Studies have shown that HOTAIR promotes development of glioma by inhibiting Mir-326 and further promoting the expression of fibroblast growth factor 1 (FGF1), which plays a significant carcinogenic role in tumorigenesis by activating the MEK1/2 and PT3K/AKT pathways (Hadari et al., 2001; Ke et al., 2015). It has been recently reported that miRNA and lncRNA control the occurrence and development of glioma, and it is proposed that the interaction of miRNA-lncRNA may provide new insights into the identification of therapeutic targets of glioma (Angelopoulou et al., 2020; Wang et al., 2021). The level of expression of miR-148b-3p, belonging to the miR148/152 family, decreased in several tumor cell lines. In addition, miR-148b-3p was also demonstrated to bind to HOTAIR in a sequence-specific manner (Sa et al., 2017). Downregulation of the expression of miR-326 was observed in glioma specimens and glioma cell lines (U87 and U251), which correlated negatively with the pathological grade of glioma Studies have shown that HOTAIR inhibits miR-326 and promotes the expression of the fibroblast growth factor 1 (FGF1) to promote the development of glioma. FGF1 plays a significant carcinogenic role in tumorigenesis by activating the MEK1/2 and PT3K/AKT pathways (Wang et al., 2016a).

To sum up, miRNA can be potentially used for the diagnosis of glioma. MiRNA-21 has been proved to be the most reliable marker for the diagnosis of glioma. A novel miR-301a/ZNRF3/wnt/β-catenin signaling feedback loop that serves critical roles in glioma tumorigenesis, and that may represent a potential therapeutic target. Targeting KCNQ1OT1 may represent a promising strategy in glioma therapeutics. However, the treatment applicability of miRNA is limited as it exhibits poor stability and low efficiency under conditions of targeted delivery. However, progress has been made in recent years in the field of the development of nano carriers. MiRNAs can be enclosed inside nano capsules to improve their stability and achieve targeted delivery. This can block the delivery of nutrients to the tumors and effectively inhibit the growth of tumors. Thus, new ideas for the identification of therapeutic targets of gliomas can be obtained. MiRNA can effectively help in the diagnosis and treatment of glioma. However, to date, preliminary experiments have been conducted. However, with the progress of scientific research, the accuracy of the targeted delivery method and the drug loading rate or organic or inorganic nanoparticle carriers can be improved. It is noteworthy that the drug-releasing property is controllable. The process of nano drug loading can also block the key factor activation pathway associated with the signal pathway to limit the further occurrence and development of tumors. We believe that the results can help us gain a deeper understanding of gliomas. The results can also help to identify new treatment methods to gradually address the global problem posed by tumors.

## HGF/MET Signal Pathway

### Expression of MET and HGF in Glioblastoma

More research has shown that MET and its ligand HGF play an important role in proliferation, survival, migration, invasion of angiogenic stem cells, drug resistance and recurrence of glioblastoma cells (Guo et al., 2017). The loci of human MET proto-oncogene is located on chromosome 7q31, and HGF on chromosome 7q21.1 (Saccone et al., 1992; Parikh et al., 2014). Analysis of TCGA data demonstrated that about 30% of glioblastomas showed over-expression of HGF and MET (Mulcahy et al., 2020). Immunohistochemical staining showed that MET was located in cytoplasm and cell membrane, and there was a high expression of MET in tumor cells, blood vessels and necrotic regions of glioma samples, and the high expression intensity of MET was correlated with WHO grade of glioblastoma, shorter PFS and OS of patients suffering from glioblastoma (Petterson et al., 2015; Yu et al., 2021). Analysis of MET gene 7Q31.2 indicated that, high expression was found in 47% of primary GBM and 44% of secondary GBM (Pierscianek et al., 2013), the above data suggest that genetic alteration plays a certain role in the pathogenesis of both glioblastoma subtypes. In addition, the activation mutation of MET is an important promoter of the transformation of from low-grade glioma to secondary glioblastoma (Hu et al., 2018). Increased MET in diffuse astrocytoma was associated with shorter OS time. MET was over-expressed in 23% of unamplified glioblastomas, and the range of staining intensity was mainly from weak to moderate (Burel-Vandenbos et al., 2017). In addition to autocrine HGF, paracrine HGF can also promote the invasion of glioma and enhance the chemotactic invasion and proliferation of the MET-positive cells (Yamamoto et al., 1997). In the meantime, HGF also acts as a chemokine of microglia and may be related with the infiltration of glioma (Badie et al., 1999). All of the above mechanisms may stimulate the highly invasive progression of gliomas.

### Crosstalk Between MET and Other Signal Pathways

**FIGURE 5:**
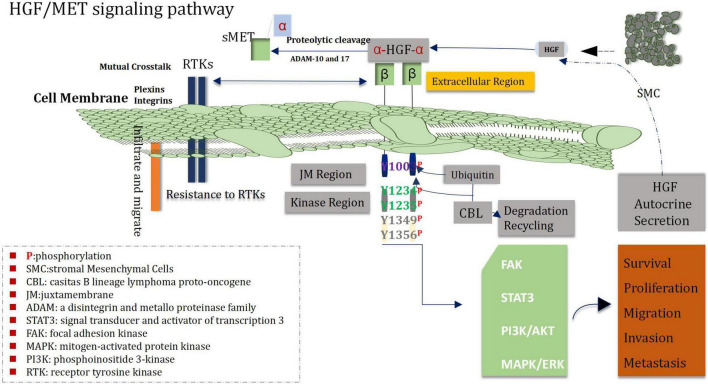
HGF/MET signal pathway.

Recent studies have found that the interaction between the HGF/MET signaling pathway and other signaling pathways play a profound and significant roles in the pathogenesis of glioblastoma (Cheng and Guo, 2019; Moosavi et al., 2019). The Wnt/β-catenin RAS/MAPK PI3K/Akt and STAT pathways which belong to downstream signal transduction medium of HGF/MET signal can enable to mediate a variety of behaviors of glioblastoma cells, including the progression of the cell cycle, invasion, dryness, angiogenesis, migration, drug-resistant, and recurrence (Zhang Y. et al., 2018; Figure 5). Both Wnt/β-catenin and HGF/MET signaling pathways regulate proliferation, migration and stem cell behavior of glioblastoma cells *via* increasing phosphorylation of β-conjunction (Y142) and Snail/Slug expression (Mimeault and Batra, 2011; Náger et al., 2015). In addition, the COX-2/PGE2 signaling pathway can influenced most markers of cancer and activate the PGE2-dependent downstream pathways (such as Ras-MAPK) (Majchrzak-Celiñska et al., 2021). It has been shown that HGF/MET signaling can promote growth and migration of gliomas cells *via* up-regulating COX-2 expression and stimulating the release of PGE2 (Zhao et al., 2015). Heat shock protein 90 (HSP90) plays a key role in protein folding, stabilization and degradation. A cancer study by Greenall et al. (2015) demonstrated that the expression of MET receptor is dependent on HSP90 protein. Therefore, inhibitors of HSP90 can prevent the growth and migration of glioma cells though inhibiting the expression of MET receptors. HGF/MET signaling is also involved in crosstalk with EGFR HER3 and EGFRvIII, which can lead to enhance activation of oncogenic signaling in glioblastoma. In addition, EGFRvIII is able to induce trans-activation of JNK2 in glioblastoma cells and then to promote increase of invasion of cell by stimulating the HGF/MET signaling circuit (Saunders et al., 2015).

### MET Receptor Is a Potential Therapeutic Target

Dysregulation of MET signal is closely associated with the tissue grade, treatment resistance, tumor recurrence and poor prognosis (Vengoji et al., 2019). Hence, the receptor of MET signal is considered as an potentially attractive target for treatment. The humanized anti-HGF monoclonal antibody YYB101 can inhibit the growth of tumors *in vitro and in situ* mouse model of human glioblastoma (Kim and Heo, 2021), Importantly, it can downregulate effective factors of cell molecular including such as p-FAK, p-met, Ki-67, pGab1, MMP2, and uPA/plasminogen (Kim H. K. et al., 2017). Onartuzumab is an anti-MET antibody, preclinical trials shown that it can inhibit the growth of glioblastoma. Similarly Crizotinib can inhibit structure-dependent tyrosine kinases, such as ROS proto-oncogenes 1 and ALK (Junca et al., 2017). Crizotinib can also effectively inhibit the survival and proliferation of MET-positive glioma stem cells, meanwhile, and can prolong survival of mice with MET-positive GSCs. However, for MET -negative GSCs, it is invalid (Tasaki et al., 2016).

Thus, we speculate that the inhibitors of the MET receptor could be a potentially promising target for treatment of glioma. At present, there are many studies on interference of HGF/MET signal in pre-clinical experiments. It is a key factor that we need to pay much closer attention to the crosstalk between the signals and then to bit by bit conquer every aspect of glioma, including mechanism, signal pathway, mutation, BBB, BTB, immune system, drug-resistant, exosome, and so on.

## Wnt/β-Catenin Signaling Pathway

### Wnt Signal Transduction

**FIGURE 6:**
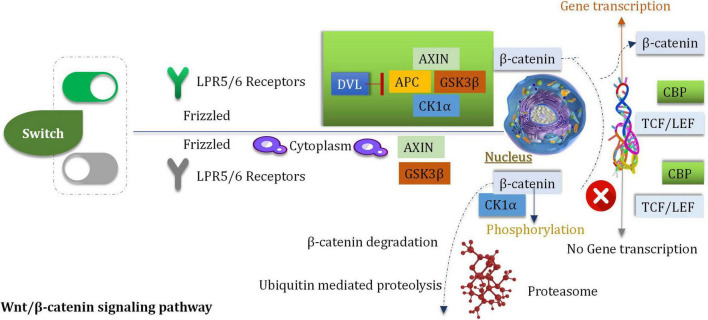
On/OFF WNT/β-catenin signal pathway.

Wnt is a member of a large family of cysteine-rich secretory glycoproteins. At least, 19 members are found in humans (Jhanwar-Uniyal et al., 2013). During the process of development, wingless/integrated (Wnt) is involved with the processes of migration, cell differentiation and proliferation (Bahmad et al., 2018). The process of Wnt signal transduction is carried out in extracellular and intracellular systems (Roussel and Hatten, 2011). The Wnt/β-catenin pathway is a classic pathway that proceeds *via* the activation of the Wnt target genes which can regulate the processes of cell differentiation and proliferation and can control stress reaction (Sheng et al., 2021). The procedures are mediated by β-catenin/T cytokine systems. The Wnt/β-catenin pathway is associated with tumorigenesis (Liu M. et al., 2017). It has been reported that the downregulation of the Wnt inhibitor-1 is observed in the abnormal Wnt signaling pathways in 75% of glioma cells (Wang et al., 2018). To date, a minimum of 11 crimp protein (Fz)-based transmembrane proteins have been identified as the receptors of the Wnt ligands (Bai et al., 2018). The pathway of Wnt signaling is initiated by after the Wnt ligand binds to the target FZ, the co-receptor, and the low-density lipoprotein receptor associated with the protein LRP5 (or LPR6) (Dai et al., 2018). Nuclear β-catenin has been reported to be a marker of the active Wnt pathway, and it can supervise the gene expression of *MGMT*. β-catenin is the primary mediator of the Wnt signal transduction process (Dai et al., 2018). It can activate the downstream target genes (such as c-Jun, c-Myc, c-fos, cyclin D1, fra-1, and those belonging to the AP1 family members) (Koch et al., 2005), which are primarily associated with the regulation of cell attribution and proliferation. The downstream target genes are activated by the interaction of β-catenin and T cytokine/LEF transcription factors in the nucleus (Dai et al., 2018; Figure 6).

### Highly Expressed β-Catenin

In glioma tissues, the expression of β-catenin was significantly higher than normal tissues (Du et al., 2020). The primary signal pathway associated with the process of glioblastoma cell invasion (induced by ionizing radiation) is the Wnt/β-catenin pathway (Dong et al., 2015). Inhibition and stimulation of the Wnt/β-catenin signaling pathway can affect the cell cycle (Gao et al., 2017). Some experiments indicated that inhibitors can reduce the proliferation and survival extent of the U87 glioma cells (Gao et al., 2017). The over-expression of Frizzled2, Wnt2, β-Catenin, and Wnt5a are observed in gliomas (Pu et al., 2009). Wnt2 and β-catenin (the key mediator) were knocked down by siRNA in the human glioma U251 cells. Under these conditions, cell proliferation and invasion were obviously inhibited, and apoptotic cell death was induced (Pu et al., 2009). In addition, the growth of tumor was delayed when nude mice which be subcutaneously inoculated with U251 glioma were treated with siRNA targeting Wnt2 and β-catenin. *In vitro and in vivo* studies were conducted, and the results revealed that the down-regulation of Wnt2 and β-catenin could be attributed to the decrease in the PI3K/p-Akt expression levels. The finding indicated that Wnt/β-catenin was interacted with the PI3K/Akt signal (Pu et al., 2009). The intracranial transplantation model of glioblastoma mice was studied, it was observed that the inhibition of Wnt5a activity could prevent the process of brain invasion and enhance the survival rate of the host (Li et al., 2021).

### WnT/β-Catenin Can Regulate O6-Methylguanine-DNA Methyltransferase

Temozolomide (TMZ) is a first-line key clinical drug for postoperative glioma. The significant drug-resistant could be attributed to the existence of *MGMT* (Oldrini et al., 2020). It has been confirmed that *MGMT* methylation combined with TMZ can be effectively served as the treatment of glioma. The postoperative survival time can also be significantly prolonged. It has also been found that Wnt/β-catenin can regulate the expression of the *MGMT* gene and reduce the drug-resistant of the tumor cells (toward chemotherapeutic drugs) (Bi et al., 2018). The approach provides a new treatment idea for clinical treatment.

Therefore, we believe that a combination of drugs which are used in chemotherapy (TMZ and Wnt/β-catenin inhibitor) can enable to prolong the survival time of postoperative of glioma. This may be a promising target for the treatment of glioma.

## Notch Signaling Pathway

### Cytoplasmic Receptor: Notch

Notch1, Notch2, Notch3, and Notch4 are the four homologous proteins that are displayed in mammals (Katoh and Katoh, 2020), and these can bind to Delta-like (Dll1-3 and -4) and serrated proteins (Jagged 1 and -2) (two ligand families). These ligands and receptors belong to the category of unidirectional transmembrane proteins (Fulop et al., 2019). The maintenance and development of the central nervous system depend on the proper functioning of these ligands and receptors. The origin of neurogenesis and other neural functions can be ascribed to neural stem cells (Bond et al., 2015). These stem cells are beneficial to maintain the homeostasis of the central nervous system. Therefore, genetic changes and functional features of neural stem cells can potentially cause occurrence of brain tumors, such as glioma (Achanta et al., 2010). The failure of treatment methods of glioma and the recurrence of glioma can be attributed to the existence of a small number of tumor cells which are known as glioma stem cells, in the microenvironment. According to reports, the glioma stem cells are identical in characteristics and invasive phenotype characterize to stem cells (Ma et al., 2018). A highly active Notch signal is observed in GSCs (Yi et al., 2019). Inhibition of the process of differentiation is observed, and stem cell-like properties are maintained. It is a of importance reason that glioma can enable to occur and resist to conventional treatment.

### Extracellular and Intracellular Notch Signaling Transduction Pathways

In the course of evolution, Notch signal pathway always presents a relatively conserved and stable condition. But, it plays a certain significant role in cell proliferation, apoptosis, stress regulation, differentiation, and angiogenesis. It can stimulate a cascade reaction and generate a series of biological effects (McIntyre et al., 2020). The notch can promote its proliferation by inhibiting the differentiation process associated with neural stem cells (An et al., 2020). During cell interaction period, notch can be transcribed when it combined with ligands.

### Notch1-Sox2 Regulates GSCs

**FIGURE 7:**
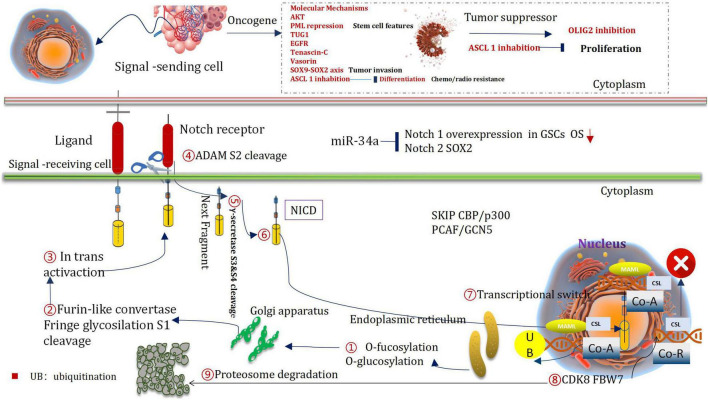
He regulatory mechanism of notch in giloma.

The levels of proteins and mRNA in Notch1, Dll1, Notch4, Dll4, Hey1, Jagged1, CBF1, Hey2, and Hes1 in glioma cells are higher expression than those in healthy brain cells. These phenomena could be attributed to the increased expression levels of pAKT and VEGF and the decreased *PTEN* level (Bazzoni and Bentivegna, 2019). The low overall survival could be ascribed to the over-expression of Notch1 (Han et al., 2017). It was also observed that the expression of Notch1 exist in GSCs around the tumors (Biswas and Rao, 2017). At the same time, the neural stem cell transcription factor Sox2 was up-regulated to reduce the methylation level of Notch1 promoter and enhance the expression of Notch1 in GSCs (Wang C. et al., 2019). The Notch4 and Notch1 levels were, respectively, associated with vimentin and GFAP (Dell’Albani et al., 2014). The expression level of Notch4 increases with the increase of the grade and primary tumor (Dell’Albani et al., 2014). The level of expression of Notch2 (in glioblastoma) could be positively correlate with the neural stem cell gene (SOX2), glial fibrillary acidic protein, vimentin, and anti-apoptotic protein (Wang J. et al., 2019). However, the expression level is negatively correlate with the pro-apoptotic protein (Dell’Albani et al., 2014; Figure 7).

Based on the transduction and regulation mechanism of the Notch signal pathway associated with extracellular and intracellular processes, we can speculate that Notch 1 and Sox2 are positively regulated by a feedback loop. It can also be hypothesized that these are related to the poor prognosis of glioma. It can further inhibit the invasion of GSCs. At the same time, based on the characteristics of over-expression of Notch4 (associated with the increase in the degree of tumor malignancy), it can be considered as the basis of classification for glioma. Thus, not only we can pay much closer attention to clinically treat glioma stem cells but also regard notch as an effective index of evaluation prognosis. A valuable indicator is of importance, which can assist us to formulate the future direction of glioma treatment.

## Hedgehog Signaling Pathway

### SonicHedgehog Signaling Pathway and GLI

**FIGURE 8:**
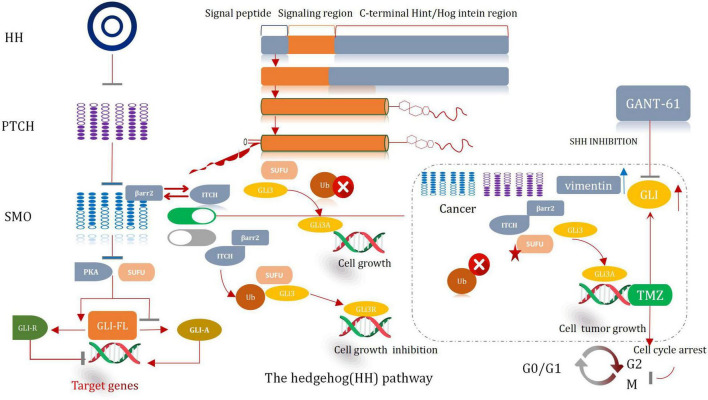
Hedgehog signal pathway.

The hedgehog (HH) signal significantly influences the process of development of organs and tissues (Finco et al., 2015). It was prove to be pivotal in a mitogen and (or) Morphological characteristics. It also plays a significant role in differentiation factor. Except it can help to maintain tissue conditions. The HH pathway presents significant lower-activity state in adults (Petrova and Joyner, 2014). The abnormal activation of the protein cause the development of several malignancies in humans, including glioma. Hence, it is a very effective therapeutic target for the treatment of cancer (Skoda et al., 2018). The major participants associated with the HH pathway include HH ligands sonic (SHH), Patched transmembrane receptors (*PTCH1* and 2), Indian (IHH) or Desert Hedgehog (DHH), glioma-associated oncogene transcription factors (*GLI1, GLI2*, and *GLI3*), and G protein-coupled receptors, such as Smoothened (*SMO*) (Asiabi et al., 2021). Typically, HH signal is activated when the HH ligand binds with PTCH1, then cause the release of SMO. Subsequently, the SMO receptor is transposed to the primary cilia and then initiates the signal cascade reaction, which eventually results in the dissociation of GLI from the negative regulator SUFU. It also causes its subsequent nucleation. The activated form of the GLI factor raises the transcription of the Hh target genes (i.e., *CCND2, BMI1, MYCN*, and *VEGF*) (Huang D. et al., 2018). Thereby, the path of cell survival, invasion, and angiogenesis are regulate throughout the entire sequential procedures. The processes correlative with the self-renewal of stem cell and the process of epithelial mesenchymal transformation (EMT) are also regulated and controlled (Figure 8; Infante et al., 2018).

### Sonic Hedgehog Inhibitor Cooperates With Temozolomide

An abnormal HH signaling pathway is observed in glioma cells. It is especially true for the Shh signaling pathway (Gampala and Yang, 2021). Somatic mutations and changes in the copy number in key genes associated with the Shh signaling pathway are observed in most of the patients suffering from glioma (Garcia-Lopez et al., 2021). These mutations are accompanied by the deletion or loss of function of *PTCH1* or *SUFU*. These are also accompanied by mutations that activate *GLI1* and *SMO*, or *GLI2* amplification (Northcott et al., 2017). In some cases, the *MYC/MYCN* gene which is responsible for transcriptional regulation is repeatedly amplified (Infante et al., 2018). The changes in the genetic characteristic result in the ligand-independent activation of the HH pathway. This promotes tumorigenesis and further worsens the condition. A number of experimental investigation have manifested that the inhibition of the Shh pathway can enhance the chemotherapeutic effect of TMZ, reduce GSCs, and even prevent the growth of GSCs, and cause tumor proliferation, differentiation, and migration (Ming et al., 2017).

Based on the recent findings of many studies, we believe that the inhibition of the HH pathway, especially the Shh signaling pathway (attributable to Shh inhibitors), can reduce the extent of tumor proliferation and then promote apoptosis. It can also strengthen the chemotherapeutic effect of the first-line clinical drug TMZ. Multi-targeted drug combination can control and reduce the recurrence and deterioration of tumors, and then prolong the survival time of patients in the near future. To be sure, this therapeutic effect is positive and worth looking forward to.

## TGF-β Signaling Pathway: Progress Made

### Inhibitors of TGF-β

TGF-β is a type of cytokine with a molecular weight of 25 kDa, which regulates the processes of cell proliferation and differentiation (Morikawa et al., 2016). It can be secreted by various types of cells, such as immune cells and nausea tumor cells. It also can regulate various biological functions, such as cell proliferation, migration, embryonic stem cell differentiation, and immune surveillance (Yang et al., 2010). The binding of TGF-β to its receptor can initiate the SAMD signal cascade (Held-Feindt et al., 2003). In many malignant tumors, such as intracranial tumors, gastrointestinal tumors, respiratory tumors, and urinary tumors, the expression of TGF-β is associated with the grade and degree of malignancy (Kjellman et al., 2000). It has been clinically proven that the TGF-β signal pathway which is interrupted is an effective strategy which can restore the antitumor immune function of glioma. LY2109761, an inhibitor of TGF-βR1 kinase, can improve the sensitivity of glioma cells toward radiotherapy. The most promising drug is Trabedersen, which is an oligoclonal nucleotide that can antagonizes TGF-βmRNA (Infante et al., 2018). It can play an inhibitory role by down-regulating TGF-β2 mRNA and can enhance the 2-year survival rate of patients suffering from late-stage glioma (Zhang et al., 2011).

### MNK1 Increases the Translation of SMAD2 mRNA

**FIGURE 9:**
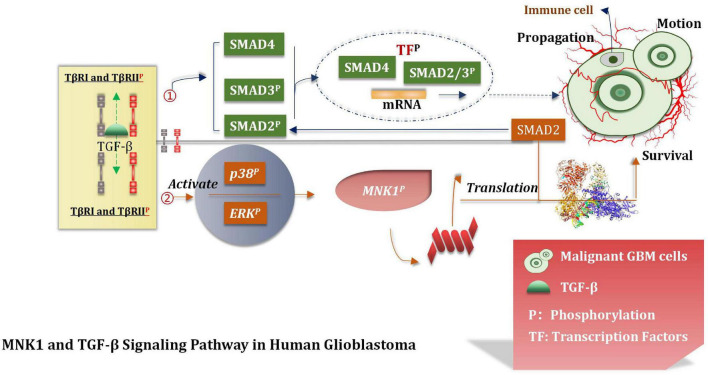
TGF-β signal pathway. TGF-β pathway interacts with TF to induce the expression of GBM cell proliferation, motility and survival genes, and participates in the translation of tumor mRNA.

Over-expressed and activated mitogen-activated protein kinase interacting kinase 1 (MNK1) is a potentially attractive and promising therapeutic target (Hou et al., 2012). This can increase the extent of translation of SMAD2 to promote the TGF-β pathway through the SMAD2/3/4 complex (Wang et al., 2016b; Mao et al., 2020). The TGF-β pathway interacts with the transcription factors (TF). This interaction induces the expression of various genes which are interrelated with the movement, proliferation, and survival of malignant GBM cells (Northcott et al., 2017). Atypical pathways can also be activated by the overactivated TGF-β receptors (TβRI and TβRII). This can lead to the phosphorylation of ERKs and p38 kinases. MNK1can further increase the extent of translation of specific mRNAs which are closely correlated with the process of cancer progression and can also be activated under these conditions (Northcott et al., 2017). It has been shown and reported that primary glioblastoma and glioma cell lines contain overexpressed MNK1 (Grzmil et al., 2011). The mRNA related with the regulation of the TGF-β pathway was identified by conducting microarray analysis of total RNA and polychromatic RNA obtained from MNK1-depleted GBM cells (Grzmil et al., 2011). After activating SMAD2/3/4, TGF-β promotes LIF transcription, thus activates the JAK/STAT3 signal pathway and then improves the proliferation ability of the stem cells. TGF-β can also induce the expression of the DNA binding inhibitory protein 1 (ID1) in GSCs and enhance the malignant proliferation of cells (Infante et al., 2018). The MNK1 signaling pathway affects and controls mRNA translation, TGF-β-induced cell movement, and vimentin expression. Results from tissue microarray analysis revealed a positive correlation between MNK1 and SMAD2 immunohistochemical staining. Grzmil et al. (2011) reported that SMAD2 is a of importance component associated with the TGF-β signaling pathway. They provided an insight into the MKN1 pathway and the mode of control of the translation process associated with the cancer-related mRNA, including SMAD2. In addition, they also suggested that the MNK1-controlled translation pathway should be used to develop targeting strategies which can even be used to effectively treat GBM (Figure 9).

A series of biological behaviors (such as over-expression of MNK1) and the increase of the translation properties of MNK1 were deeply studied. The proliferation of malignant GBM cells was also studied, and based on the results, we hypothesized that MNK1 could be used as a marker for predicting tumors. At present, eFT508 is considered as an efficient and highly selective inhibitor of MNK1 during experiments (Jin et al., 2021). But further deeper researches and more trails are needed to conduct and confirm whether inhibitors of MNK1 can be applied to the stage of clinical experiment. We believe that MNK1 is an attractive and promising therapeutic target in the near future.

## Janus Kinase/Signal Transducer and Activator of Transcription Signaling Pathway

The Janus kinase/signal transducer and activator of transcription (JAK/STAT) signaling pathway is considered as one of the central communication nodes in the cell function (Darnell, 1997). The JAK/STAT signaling pathway has profoundly influenced recent understanding attained of human health and disease. The JAK/STAT signaling pathway is evolutionarily conserved. It is composed of ligand-receptor complexes, JAKs, and STATs. There are 4 members in the JAK family: JAK1, JAK2, JAK3, and TYK2. The STAT family comprises seven members: STAT1, STAT2, STAT3, STAT4, STAT5a, STAT5b, and STAT6 (Xin et al., 2020). The composition of the JAK/STAT pathway is as follows: one is the classical JAK/STAT signaling pathway, another one is the non-classical JAK/STAT signaling pathway. Many researches have reported the importance of this pathway in malignancies (Aaronson and Horvath, 2002; O’Shea and Plenge, 2012; Ivashkiv and Donlin, 2014; O’Shea et al., 2015; Banerjee et al., 2017; Villarino et al., 2017). Thus, inhibiting the JAK/STAT pathway is promising for treating various diseases. Currently, many JAK inhibitors have achieved efficacy in many clinical settings, and more medications are currently being studied, such as STA-21, LLL-3, LLL12, and curcumin etc. (Ashrafizadeh et al., 2020; Xin et al., 2020; Shi et al., 2021). There are two ways of the regulation in JAK/STAT signaling pathways, that are positive and negative (Hu et al., 2021). The resent research shown that YTHDC1-mediated VPS25 regulates cell cycle by targeting JAK-STAT signaling in human glioma cells. VPS25 was upregulated in glioma tissues, which was correlated with poor prognosis in glioma patients. Furthermore, VPS25 knockdown inhibited the proliferation, blocked the cell cycle, and promoted apoptosis in glioma cells. Meanwhile, VPS25 induced a G0/G1 phase arrest of the cell cycle in glioma cells by directly mediating p21, CDK2, and cyclin E expression, and JAK-signal transducer and activator of transcription (STAT) activation. These results suggest that VPS25 is a promising prognostic indicator and a potential therapeutic target for glioma (Zhu et al., 2021).

### Signaling Cross-Talk Between Janus Kinase/Signal Transducer and Activator of Transcription and Other Pathways

**FIGURE 10:**
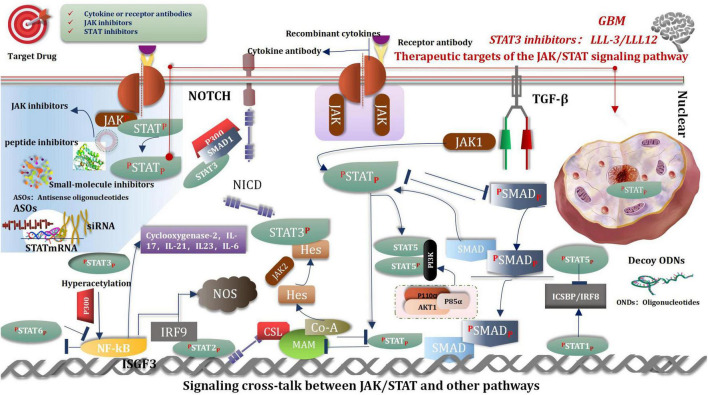
JAK/STAT signaling pathway.

Signaling cross-talk between JAK/STAT and other pathways is an intricate and colossal network, happens at different levels, and covers varieties of molecules, such as a ligand, receptor, phosphorylation, JAK, STAT, and gene transcription factors etc. (Zhu et al., 2021). These cross-talk activities play crucial roles in pluripotency and differentiation transcription program, immune regulation, and tumorigenesis. For example, TGF-β hedges IL-12 mediated JAK2 and TYK2 tyrosine phosphorylation (Bright and Sriram, 1998). Notch signaling inhibits JAK/STAT activation by interfering with STAT translocation to the DNA domain, and signals of JAK/STAT inhibited Notch signaling conversely (Assa-Kunik et al., 2007). SMAD3 inhibits STAT3 activation *via* recruiting PIAS3 to STAT3 and so on (Wang et al., 2016a; Hu et al., 2021; Figure 10).

According to the above analysis, the future studies should offer innovative insights into the potential mechanisms of the JAK/STAT pathway effects and cancer development. Moreover, we should concentrate all efforts on maximize efficacy and minimize side effects in patients in different stages of certain tumors and to explore more biomarkers that predict efficacy and offer prognoses.

## Low-Grade Glioma as Neurodevelopmental Disorders and Mitogen-Activated Protein Kinase Signaling Pathway

**FIGURE 11:**
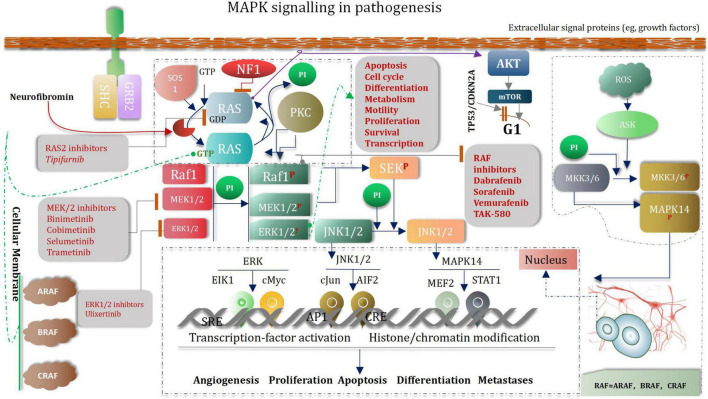
MAPK signaling pathway.

Pediatric low-grade glioma (the most common glioma is the WHO grade I pilocytic astrocytoma PA) is the most common CNS tumor of childhood. Although overall survival is good, disease often recurs (Fangusaro et al., 2019). Abnormal MAPK pathway activation is the most common genetic aberration in pediatric low-grade glioma (Hargrave, 2009; Jones et al., 2013, 2018). Several drugs that target the MAPK pathway have been developed (Zhang et al., 2013). Selumetinib is a selective and potent orally available non-ATP-competitive small molecule inhibitor of MEK1/2 (Fangusaro et al., 2019). To our knowledge, aside from the use of mTOR inhibitors in tuberous-sclerosis-associated subependymal giant cell astrocytoma, 33 which is rare in the pediatric population, selumetinib is one of the first prospectively tested and active molecularly targeted agents in pediatric low-grade glioma (Fangusaro et al., 2019). MAPK (mitogen-activated protein kinase) signaling pathways regulate a variety of biological processes through multiple cellular mechanisms (Yue and López, 2020). MAPK cascade is a critical pathway for human cancer cell survival, dissemination, and resistance to drug therapy (De Luca et al., 2012; Elbaz et al., 2012). The MAPK/extracellular signal-regulated kinase (ERK) pathway is a convergent signaling node that receives input from numerous stimuli, including internal metabolic stress and DNA damage pathways and altered protein concentrations, as well as through signaling from external growth factors, cell-matrix interactions, and communication from other cells (Morrison, 2012; Yang et al., 2013). Mutated genes responsible for the regulation of cell fate, genome integrity, and survival can lead to increased protein amplification and alter the tumor microenvironment, thus overactivating the pathway (Birkbak and McGranahan, 2020). These mutations can occur upstream in membrane receptor genes, such as epithelial growth factor receptor (EGFR) (Wang et al., 2020) in signal transducers (RAS) (Malumbres and Barbacid, 2003) regulatory partners (Sprouty) (Muram-Zborovski et al., 2010; Yan W. et al., 2020) and downstream kinases that belong to the MAPK/ERK pathway itself (BRAF) (Fang and Richardson, 2005; Burotto et al., 2014; Moon and Ro, 2021). Low-grade glioma growth control pathways. LGGs arise from mutations in genes whose protein products regulate RAS pathway signaling. In this manner, mutations in genes encoding receptor tyrosine kinases (RTK), which transduce extracellular signals, as well as the downstream effectors PTPN11, RAS, BRAF/RAF, lead to hyperactivation of mitogenic signaling downstream through the MAP kinase and PI3-kinase pathways. In addition, mutational inactivation of the neurofibromatosis type 1 (NF1) tumor suppressor gene results in increased RAS activity. MEK and AKT can shorten G1 transition through mammalian target of rapamycin (mTOR)-dependent and -independent signaling. The presence of stromal growth factors and chemokines in the tumor microenvironment generate growth-promoting signals through tumor cell receptors and further enhance glioma expansion in a paracrine fashion (Baker et al., 2016; Figure 11).

## Blood–Brain Barrier and Signal Pathways

The blood–brain barrier (BBB) is an extremely of significance factor to consider when determining treatments for various neurological diseases, both because disruption of the BBB can result in grievous pathology observed in many different diseases, especially brain tumors, but also because crossing the BBB is a basic element in the development of Central nervous system therapy (Daneman and Prat, 2015). BBB, Genetic and transcriptomic studies have proved activation of signaling pathways, such as WNT–β-catenin and sonic hedgehog (SHH)-dependent signaling in brain ECs (endothelial cells) within the BBB (Daneman and Prat, 2015). Intriguingly, some pathways including the G protein-coupled receptor (GPCR), GPR124 and WNT-β-catenin axis also regulate additional characteristics of vascular structure and function (Kuhnert et al., 2010; Umans et al., 2017; Griveau et al., 2018) CNS (central nervous system) ECs (endothelial cells) signaling pathways can be directly regulated by pericytes and astrocytes.

**FIGURE 12:**
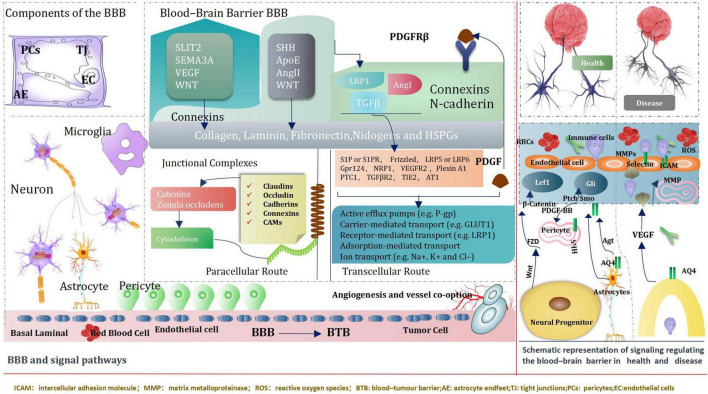
BBB and signal pathways.

Neuronal and non-neuronal cells regulate the expression of transport and tight junction proteins in ECs, which in turn may “loosen” or “tighten” the BBB (Arvanitis et al., 2020). We introduce ways of key signaling pathways connecting astrocytes, pericytes and neurons to ECs (Sweeney et al., 2019). These pathways will modify transcellular transport by changing the expression of transporters and the paracellular path by tangling junctional protein complexes. Importantly, ECs mutually adjust and control components of the NVU (neurovascular unit). For instance, cognate receptor on pericytes are stimulated by EC-secreted TGF-β (Umans et al., 2017; Griveau et al., 2018). During development and maturation, glial cells, pericytes and neurons regulate EC behavior *via* numerous ligands and receptors, which in turn activate downstream signaling cascades that instruct expression of junctional and transcytosis proteins and control CNS homeostasis. Astrocytes directly modulate NVU demands such as water content in the neuroparenchymal space *via* the major water channel protein AQP4 (aquaporin 4) (Vanlandewijck et al., 2018) regulate immune cell and cancer cell (Arvanitis et al., 2020; Figure 12).

In conclusion, the brain microenvironment can thwart the effectiveness of drugs against primary brain cancer as well as brain metastases. It is a tremendous challenges that posed by the BBB and BTB for drug delivery, how multiple cell types, signaling pathways, and signaling cross-talk dictate BBB function and the role of the BTB in tumors progression and treatment. We believe emerging signal pathway targeting molecular to improve drug delivery across the BBB and BTB (blood–tumor barrier) and improve Prognosis of giloma, such as immune checkpoint inhibitors, Nanotechology, and engineered T cells. Common key signal pathways inhibitors glioma, Table 1.

**TABLE 1:** Key signal pathways inhibitors glioma.

Type	Inhibitors	References
Hippo/YAP	NSC682769	Saunders et al., 2021
	peptide 17	Liu M. et al., 2017
	Verteporfin	Deng et al., 2022
	Cbx7	Nawaz et al., 2016
	Digoxin	Elbaz et al., 2012
	miR-376a	Deng et al., 2022
	G007-LK	Kierulf-Vieira et al., 2020
	Silibinin	Bai et al., 2018
PI3K/AKT/mTOR	p-mTORS2448	Liu M. et al., 2017
	Apitolisib (GDC-0980)	Omeljaniuk et al., 2021
	POU2F2	Yang R. et al., 2021
	Carnosine	Oppermann et al., 2019
	(2-(2,4-dioxopentan-3-ylidene)hydrazineyl) Benzonitrile,	Viswanathan et al., 2019
	Gartanin	Liu M. et al., 2017
	NVP-LDE-225	Nanta et al., 2019
	NVP-BEZ-235	Nanta et al., 2019
	β-mangostin	Li et al., 2020
	RES-529	Weinberg, 2016
TSC-mTORC1	Everolimus	Trelinska et al., 2017
	G3BPs	Prentzell et al., 2021
	PQR309	Yang et al., 2020
HGF/MET	SB-hHgf.Met.ShP53	Qin et al., 2020
	SPINT2/HAI-	Kongkham et al., 2008
	Selumetinib	Ryall et al., 2020
	Trametinib	Ryall et al., 2020
	Cobimetinib	Ryall et al., 2020
	AZD4547	Ryall et al., 2020
	TLN-4601	Mason et al., 2012
PI3K/Akt	P4HA2	Lin et al., 2021
	Baicalein	Yang Y. et al., 2021
	JQ1	Wen et al., 2019
	LINC00673	Zhang F. et al., 2018
	miR-128-3p	Huo et al., 2019
	COX-2	Majchrzak-Celiñska et al., 2021
	**Annexin-A1**	Wei et al., 2021
STAT	CX-4945	Liu X. et al., 2020
WnT/β-catenin	G007-LK	Kierulf-Vieira et al., 2020
	Celecoxib	Majchrzak-Celiñska et al., 2021
	2,5-DMC, etori-, rofe-	Majchrzak-Celiñska et al., 2021
	Valdecoxib	Majchrzak-Celiñska et al., 2021
	MIR22HG	Han S. et al., 2020
	CBX7	Bao et al., 2017
	MRK003	Herrera-Rios et al., 2020
Notch	LDFI (Leu-Asp-Phe-Ile)	Panza et al., 2020
	RO4929097	Xu et al., 2016
	MRK003	Herrera-Rios et al., 2020
Hedgehog	CBL0137	Mo et al., 2021
	LDE225/Sonidegib	Rimkus et al., 2016
	GDC-0449/Vismodegib	Rimkus et al., 2016
TGF-β	LY3200882	Yap et al., 2021
	Cediranib	Pham et al., 2015
	Vandetanib	Pham et al., 2015
	COX-2	Wang C. et al., 2019
	mPGES-1 CYP4A	Wang C. et al., 2019
JAK/STAT	AZD3759	Yin et al., 2021
	Ruxolitinib	Delen and Doğanlar, 2020
	YM155	Jane et al., 2017
	WP1066	Vangala et al., 2020
	Curcumin	Hu et al., 2021
	LLL-3	Hu et al., 2021
	LLL12	Hu et al., 2021
MAPK	Trametinib	Perreault et al., 2019
	PD325901	Wu et al., 2020
sHH	NVP-LDE-225	Nanta et al., 2019
	NVP-BEZ-235	Nanta et al., 2019

## Conclusion and Prospect

In recent years, researchers have focused on signal pathways and glioma. This paper discusses the mechanisms associated with the Hippo/YAP, PI3K/AKT/Mtor, miRNA, Hedgehog, WnT/β-catenin, Notch, and TGF-β signal pathways and the key enzymes associated with glioma. The results can potentially help explore new diagnostic and prognostic biomarkers for the identification of new and efficient molecular therapeutic targets. However, further research should be conducted to clarify a few points. Significant levels of heterogeneity are observed in tumor cells (attributable to the recruitment of various cells), resulting in the generation of a tumor microenvironment. There are few reports on signal pathways and tumor heterogeneity. Though the relationship between signal pathways and glioma progression has been reported in the literature, the crosstalk between multiple signal pathways makes it difficult to determine the effective molecular targets associated with glioma treatment. We believe that the application of organic or inorganic nano carriers or exosomes (combined with clinical medication and adjuvant immunotherapy, such as monoclonal antibody-targeted therapy, polypeptide vaccine, DC vaccine, adoptive immunotherapy, and cytokine therapy), can potentially help in effectively treating glioma. Large-scale cohort studies and related molecular experiments should be conducted to find more reliable molecular therapeutic targets and address the existing problems.

## Author Contributions

HW: design of the work. MW: the acquisition analysis. QM: interpretation of data. YL: the creation of new software used in the work. HZ: have drafted the work or substantively revised it. All authors read and approved the final manuscript.

## Conflict of Interest

The authors declare that the research was conducted in the absence of any commercial or financial relationships that could be construed as a potential conflict of interest.

## Publisher’s Note

All claims expressed in this article are solely those of the authors and do not necessarily represent those of their affiliated organizations, or those of the publisher, the editors and the reviewers. Any product that may be evaluated in this article, or claim that may be made by its manufacturer, is not guaranteed or endorsed by the publisher.

## Abbreviations

PTEN: phosphatase and tension homolog deleted on chromosome ten
GBM: glioblastomas
Sav: salvador
Mats: mob as the tumor suppressors
Yki: yorkie
Yap: yes-associated protein
PI3K: phosphatidylinositol-3-kinase
PIP3: phosphomembrane-bound phosphatidylinositol-3
PI3K: phosphatidylinositol-3-kinase
Mtor: mammalian target of rapamycin
SHH: sonic hedgehog
LATS1: large tumor suppressor homolog 1
MGMT: O6-methylguanine-DNA methyltransferase
CKAP4: cytoskeleton-associated protein 4
TFA: transcription factor A
AMOTL1: Angiomotin-like 1
TMZ: temozolomide
Mst1: mammalian sterile 20-like 1
P: phosphorylation
SMC: stromal Mesenchymal Cells
CBL: casitas B lineage lymphoma proto-oncogene
JM: juxtamembrane
ADAM: a disintegrin and metallo_x0002_proteinase family
STAT3: signal transducer and activator of transcription 3
FAK: focal adhesion kinase
MAPK: mitogen-activated protein kinase
PI3K: phosphoinositide 3-kinase
RTK: receptor tyrosine kinase
RISC: RNA-induced silencing complex
FGF1: fibroblast growth factor-1
HOTAIR: hox transcript antisense RN
Sox2: sex determining region Y-box 2
MNK1: mitogen-activated protein kinase-interacting kinase 1
ID1: inhibitor of DNA binding protein-1
GALNT2: galactosaminyl transferase 2
Akt/FoxM1: Forkhead box M 1
MYBL2: MYB-related protein B2
EMT: Epithelial-mesenchymal transition
TSC: tuberous sclerosis complex
S6K: protein S6 kinase
4E-BP: 4E binding proteins
HIF1a: hypoxia-inducible factor 1a
VEGF: vascular endothelial growth factor
SGs: stress granules
Rheb: Ras homolog enriched in brain
lncRNAs: long non-coding RNAs
ZNRF3: zinc and ring finger 3
TF: transcription factors
JAK/STAT: Janus kinase/signal transducer and activator of transcription
LGG: low-grade glioma
MAPK: mitogen-activated protein kinase
ERK: extracellular signal-regulated kinase
EGFR: epithelial growth factor receptor
NF1: neurofibromatosis type 1
BBB: blood–brain barrier
ECs: endothelial cells
CNS: central nervous system
GPCR: G protein-coupled receptor
NVU: neurovascular unit
AQP4: aquaporin 4
BTB: blood–tumor barrier.
